# Disrupting arrhythmia in a professional male wrestler athlete after rapid weight loss and high-intensity training—Case report

**DOI:** 10.3389/fcvm.2023.1091603

**Published:** 2023-01-25

**Authors:** Aleksandra Milovančev, Tatjana Miljković, Aleksandra Ilić, Anastazija Stojšić Milosavljević, Milovan Petrović, Roberto Roklicer, Nemanja Lakičević, Tatjana Trivić, Patrik Drid

**Affiliations:** ^1^Faculty of Medicine, University of Novi Sad, Novi Sad, Serbia; ^2^Department of Cardiology, Institute of Cardiovascular Diseases of Vojvodina, Sremska Kamenica, Serbia; ^3^Faculty of Sport and Physical Education, University of Novi Sad, Novi Sad, Serbia; ^4^Sport and Exercise Sciences Research Unit, University of Palermo, Palermo, Italy

**Keywords:** rapid weight loss, arrhythmia, wrestling, combat athletes, heart, case report

## Abstract

**Introduction:**

Physiological heart adaptations may lead to increased susceptibility to arrhythmia in athletes. Furthermore, vigorous training and method like rapid weight loss (RWL) in combat sports could pose additional risks. This case represents how routine cardiovascular screening during high-risk methods like RWL and high-intensity training (HIT) reveal abrupt ventricular arrhythmias in a young athlete.

**Case report:**

We report a case of a 20-year-old male wrestler athlete who developed disrupting arrhythmia during RWL and HIT. The study included: a medical exam, 12 lead electrocardiograms (ECG), transthoracic echocardiogram (ECHO), and 24 h of continuous ECG monitoring in baseline, phase one (P1), (in which the athlete had to simulate RWL through vigorous training and dietary intervention and HIT) and phase two (P2), (with the same HIT protocol performed without the RWL procedure). Baseline laboratory analyses were without abnormalities, ECG showed sinus rhythm with one premature atrial contraction (PAC) and ECHO showed signs of concentric remodeling with preserved systolic, diastolic function, and global longitudinal strain. After P1 RWL simulation, he lost 5.15% of body weight in 3 days, which resulted in lower blood glucose levels, higher urea, creatinine, creatine kinase (CK), CK-MB levels, and slightly increased levels of NT pro-BNP, ECG revealed sinus rhythm with one ventricular premature beat (VPB), 24-h continuous electrocardiogram (ECG) revealed frequent ventricular premature beats (PVB) 2,150/ 24 h, with two couplets, and 8 PAC. After an advised 4-week period of de-training continuous 24 h, ECG monitoring was improved with only occasional PVB. The 24 h continuous ECG monitoring was repeated after HIT and revealed even more frequent PVB, 5% of all beats for 24 h, 4,205 in total, and almost all VPB were in bigeminy and trigeminy. The athlete was advised against RWL and extremely vigorous exercise and for regular, frequent checkups with occasional ECG monitoring during and after exercise.

**Conclusion:**

The short and long-term implication of abrupt ventricular arrhythmias provoked by intensive training and methods like RWL is unknown. We postulate that cardiovascular screening is necessitated, especially during high-risk methods like RWL and HIT, in helping us prevent adverse outcomes and come to individual-based clinical making decisions for each athlete.

## 1. Introduction

Wrestling is one of the oldest Olympic sports, with Greco-Roman and freestyle wrestling being internationally recognized as competitive forms of wrestling. As a combat sport, wrestling is exceedingly mentally and physiologically demanding (1). Rapid weight loss (RWL) is highly prevalent in wrestling (2). Many wrestlers engage in RWL before weigh-in, followed by rapid weight gain (RWG) after weigh-in/before a competition to get a real or perceived advantage over their lighter opponents. In terms of combat sports, athletes practicing RWL usually reduce ~2–10% of their body weight in a time of 1–7 days (3), but in practice, there is no universally applied RWL procedure but rather many variations of the same theme (4). For example, our study reports an athlete who loses more than 5% in 3 days. Methods to induce RWL are similar to those found in other combat sports and are centered around active and passive dehydration, gut content manipulation, and glycogen depletion (2, 5). Concerns about acute health risks from the continued use of RWL have mainly focused on the loss of more than 5% of body mass using extreme dehydration or food deprivation on days 1 or 2 before weigh-in. Health risk that may be caused by RWL depends on various factors, such as the total amount of body mass (BM) reduction, the time for this reduction, and the frequency of episodes and/or strategies used for RWL (6). Despite the documented health risks and consequences that can even lead to death (7), it is still practiced (8).

An athlete's heart physiologically adapts morphology and function to cope with the demands of exercise. These changes pose the risk of different electrical disturbances, which are the main cause of adverse cardiovascular events in athletes (9). Regular medical checkups are usually done in rest or on a treadmill with continuous electrocardiogram (ECG) monitoring. There is a gap in knowledge of whether are there heart rhythm disturbances or myocardial injuries during procedures like RWL or vigorous training.

The aim of this study was to assess the effects of 3-day RWL on rhythm disturbances and biomarkers of myocardial injury in a young male, apparently healthy wrestler. We herein describe a case of a professional wrestler athlete with acute onset of ventricular arrhythmias after RWL and high-intensity training.

## 2. Case description

### 2.1. Athlete overview

A professional male wrestler (age 20, height 177.5 cm, weight 77.5 kg) volunteered to participate after providing informed written consent and permission to publish obtained data. A wrestler had 14 years in sport, though last 7 years he has been competing in wrestling, on the national level. He usually performs RWL a couple of times a year, and loses 3–6% of his body weight in 3–5 days. The athlete was without a medical history of previous disorders. The medical exam was performed 7 days before RWL, baseline 12 lead electrocardiogram (ECG) was without abnormalities, transthoracic echocardiogram (ECHO) showed signs of concentric remodeling with preserved systolic, diastolic function, and global longitudinal strain (Table 1). Laboratory analyses were without any abnormalities (Table 2). The experiment protocol was conducted and supervised by assistants and professors of Faculty of Sport and Physical Education, University of Novi Sad. The medical assessment and interpretation were conducted by cardiologists with experience in sports cardiology.

**Table 1:** Baseline echocardiographic parameters.

**Parameters**	
LVEDd (mm)	44
LVEDd/BSA (mm/m^2^)	22.8
LVEDs (mm)	29
LVEDs/BSA (mm/m^2^)	15.03
IVS (mm)	10
PLW (mm)	10
EDV (ml)	90
EDVI (ml/m^2^)	46.63
ESV (ml)	36
ESVI (ml/m^2^)	18.65
SV (ml)	54
SVI (ml/m^2^)	27.98
RWT (mm)	0.45
LVM (g)	156
LVMI (g/m^2^)	81
Aortic root (mm)	27
Cuspis separation (mm)	21
Ascending aorta (mm)	29
EF%	60
FS%	34.1
E-wave (m/s)	0.75
A (m/s)	0.5
E/A ratio	1.5
e' sep (cm/s)	0.1
E/e'sep ratio	6.8
e' lat (cm/s)	0.12
E/e' lat ratio	6.25
e'average (cm/s)	0.11
E/e' average	6.82
LAVs (ml)	52
LAVsI (ml/m2)	26.94
GLVS (%)	−18.6
RVEDd (mm)	27
TAPSE (mm)	22
RVSP (mmHg)	29
MR	mild
TR	mild

A, late atrial contraction; E, early wave; E/A ratio, peak early wave to atrial late wave ratio; e'average, average peak early velocity; E/e' average, early wave to average peak early velocity; E/e' sep, early wave to septal peak early velocity; E/e' lat ratio, early wave to lateral peak early velocity; e' lat, lateral peak early velocity; e' sep, septal peak early velocity; E/e' lat ratio, early wave to lateral peak early velocity; EDV-LV, End-diastolic volume; EDVI-LV, End-diastolic volume/ body surface area; EF, ejection fraction; ESV-LV End-systolic volume; ESVI–LV, End-systolic volume/ body surface area; FS, fraction of shortening; GLVS- global left ventricular strain, IVS, inter-ventricular septum, LVHL, left ventricle hypertrophy level, LAVs, left atrium volume at end-systole, LAVsI, left atrium volume index; LVM, left ventricle mass; LVMI, left ventricle mass index, LVEDd, LV end-diastolic diameter, LVEDd/BSA, LV end-diastolic diameter and body surface area ratio; LVEDs, LV end-systolic diameter; LVEDs/BSA, LV end-systolic diameter and body surface area ratio; MR, Mitral regurgitation; PLW, posterolateral wall; RVEDd, Right ventricular End-diastolic diameter; RWT, relative wall thickness; TAPSE, Tricuspid annular plane systolic excursion; TR, Tricuspid regurgitation; SV, Stroke volume; SVI, stroke volume/body surface area.

**Table 2:** Comparison of analyzed baseline P1 and P 2 parameters.

**Parameter**	**Baseline**	**P1**	**P2**
**Body composition**			
Body height (cm)	177.5	177.5	177.5
Body mass (kg)	77.7	73.7	77.4
BMI (kg/m^2^)	25.2	23.8	25
Fat mass (%)	17.6	13.9	16.7
Muscle mass (%)	41.9	43.9	42.3
Visceral body fat (%)	7	6	7
Basal metabolic rate (kcal)	1,775	1,720	1,768
**Laboratory analysis**			
GLUC (mmol/l) (4.1–6.1)	6	3.9	5.8
CKI (U/L) (26–100)	226	424	486
MBI (U/L) (7–25)	28	53	19
BUN (mmol/l) (2.5–7.5)	6.8	8.3	4.3
URCA (umol/l) (208–428)	341	484	379
CRE (umol/l) (62–106)	92	100	111
LDI (U/L) (125–220)	210	327	204
AST (U/L) (5–34)	21	39	31
ALT (U/L) (5–55)	40	32	42
S-HS Troponin I (ng/L) ( ≤ 10)	10	10	10
S-NT-proBNP (pg/ml) ( ≤ 125)	12.1	26.8	8.7
WBC (10^**9**^/L) (4–11)	6.23	22.61	12.77
NEUT (10^**9**^/L) (2–7.6)	2.18	20.28	10.36
LYMPH (10^**9**^/L) (1.32–3.57)	3.18	1.17	1.55
MONO (10^**9**^/L) (0.30–0.82)	0.71	1.14	0.84
EO (10^**9**^/L) (0.04–0.54)	0.15	0.01	0.01
BASO (10^**9**^/L) (0.01–0.08)	0.01	0.01	0.01
RBC (10^**12**^/L) (4.50–6.50)	5.38	5.31	5.2
HGB (g/L) (130–170)	158	155	153
HCT (L/L) (0.400–0.540)	0.461	0.448	0.438
MCV (fL) (80.0–100.0)	85.5	84.4	84.2
MCH (pg) (27.0–32.0)	29.4	29.2	29.4
MCHC (g/L) (320–360)	343	346	349
RDW-SD (fL) (37.0–54.0)	41.2	40.2	40.2
RDW-CV (%) (11.0–16.0)	13.3	13	13.1
PLT (10^**9**^/L) (150–400)	172	202	183
MPV (fL) (6.0-11.0)	9.4	10.3	9.8
PDW (fL) (9.0–17.0)	10.6	11.7	10.3
P-LCR (%) (13.0–43.0)	20.7	26.4	22.8
PCT (L/L) (0.0015–0.0050)	0.0016	0.0021	0.0018

ALT, alanine aminotransferase, AST, aspartate aminotransferase, BASO, basophils; BL, baseline measurement; BMI, body mass index; BUN, blood urea nitrogen, CKI, creatine kinase; CRE- Creatinine, EO, eosinophils; GLUC, blood glucose level; HCT, hematocrit level; HGB, hemoglobin level; LDI, Lactate dehydrogenase; LYMPH, lymphocytes; MBI, creatine kinase-MB isoenzyme; MCH, mean corpuscular hemoglobin; MCHC, mean corpuscular hemoglobin concentration; MCV, mean corpuscular volume; MONO, monocytes; MPV, mean platelet volume; NEUT, neutrophils; P1, phase one (rapid weight loss & high intensity training); P2, phase two (high intensity training); PCT, procalcitonin; PDW, platelet distribution width; P-LCR, platelet-large cell ratio; PLT, platelet; RBC, red blood cells; RDW-CV, range of variation of red blood cell volume that is reported as a part of a standard complete blood count; RDW-SD, differences in the volume and size of red blood cells (standard deviation); S-HS Troponin I, high sensitivity cardiac troponin; S-NT-proBNP, NT-pro B-type Natriuretic Peptide; Potassium, potassium concentration; S-Sodium, sodium concentration; URCA, Uric acid, WBC, white blood cells.

This study was conducted in accordance with the Helsinki declaration. Ethical approval was obtained from the ethics committee of the University of Novi Sad, Novi Sad, Serbia (Ref. No. 46-06-02/2020-1).

### 2.2. Protocol (dietary, training)

**Figure 1:**
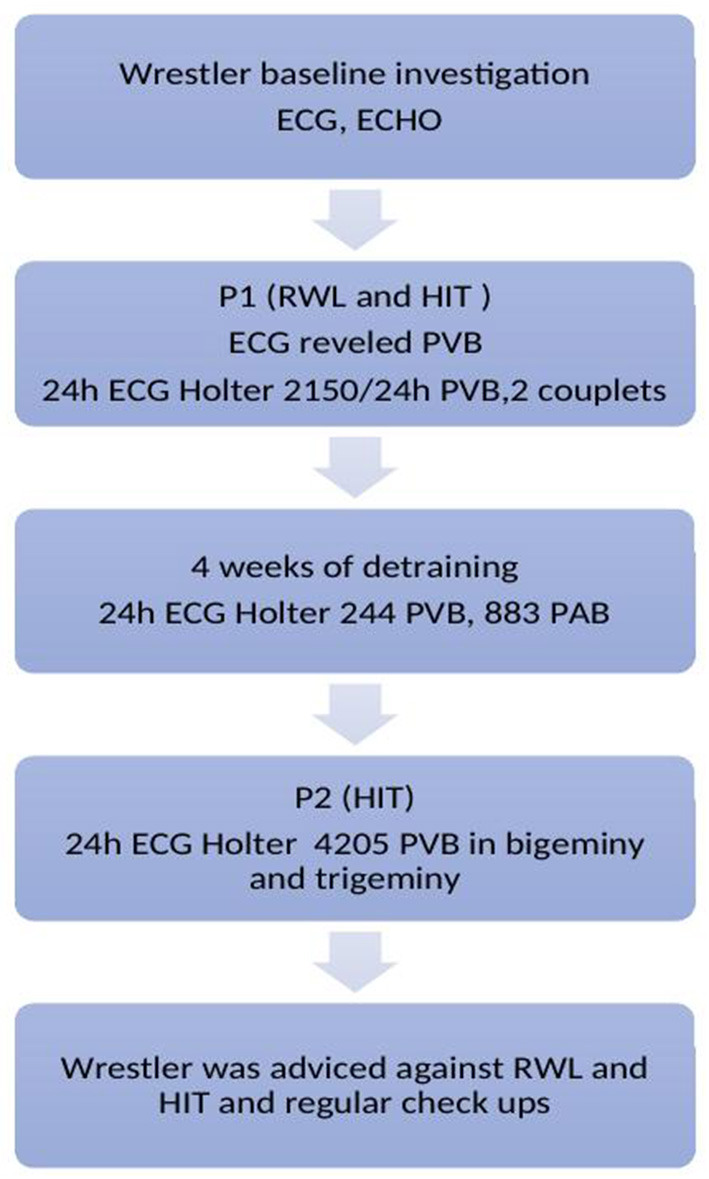
Timeline legend. ECG, Electrocardiogram; ECHO, Echocardiography; HIT, High intensity training; PVB, Premature ventricular beats; PAB, Premature atrial beats; RWL, Rapid weight loss.

This study included two phases (Figure 1, timeline). Phase one (P1), in which the athlete had to lose 5% of his body in 3 days through vigorous training and dietary intervention. The day of training (testing) was the last day of the RWL period, after which the measurements were performed. In Phase two (P2), the same high-intensity training protocol was performed with no RWL procedure included. The total duration of the training was 90 min. The respondent was familiar with the testing procedure and was instructed to perform the protocol with the same sparring partner on both periods (P1 and P2).

#### 2.2.1. Warm-up

Participant started with a warm-up that consisted of 5 min of foam rolling, followed by 5 min of dynamic stretching. Standard and sport-specific warm-ups were conducted for the next 7 min after which 8 min of gymnastic and acrobatic elements were performed by wrestler (total warm-up time = 25 min).

#### 2.2.2. Testing protocol

The main part of the training consisted of an intensive throwing technique (15 s) interspersed with low-intensity aerobic running (45 s). In fact, the participant initially began a low-intensity circular run (45 s). Five seconds before the throwing part of the test, the respondent was instructed to place himself at a point 9 m away from his sparring partner. At the sound signal, he had to run to his sparring partner as fast as possible and perform the throwing technique. Immediately after the throw, as fast as possible, he had to return to the starting point of 9 m. Participant had to perform at least 4 shoulder throws together with sprints during this period of 15 s. When the throwing part was completed, respondent started a new set of low-intensity running. After the subject completed the set of 10 min, a 3-min break was applied. Four sets of the explained protocol have been realized (total duration of testing protocol = 40 min).

#### 2.2.3. Cool down

At the end of the test protocol, the cool down phase was employed. This phase consisted of 12 min of low-intensity circular running.

### 2.3. RWL procedure

#### 2.3.1. First day

After the baseline assessment (in the morning) the respondent implemented the following meal and training plan. The subject had one can of tuna for lunch. Furthermore, plenty of fluid during the rest of the day was consumed (lemon and grape juices, and a lot of water ingestion). Prior to the evening training session, participant performed a low intensity activity (active rest) as walking, after which he attended the regular wrestling training session. For dinner, the respondent had a meal consisting of one green apple.

#### 2.3.2. Second day

On the second day, many caffeine rich drinks were consumed. The first beverage in the morning was a cup of green tea. A glass of squeezed ginger, lemon and grape was the second beverage ahead of the morning workout. The training session in the morning included 45 min of low intensity aerobic running. For lunch, the respondent had a meal consisting of slice of fish, half portion of rice, and lettuce. During the afternoon, the subject rested until the evening training session with no more fluid intake on this day. The training session in the evening consisted of low intensity running (45 min of jogging interspersed with gymnastic and acrobatic elements) in a plastic suit (in order to induce extensive sweating). Immediately after the training, the respondent used sauna-−3 sets of 5 min spent in heated environment with 2–5 min break (spending time outside the sauna).

#### 2.3.3. Third day

On the final day, the above explained training session was performed in the morning. No liquids were consumed prior and/or during the test protocol (before the final measurement). 2 h after the procedure, weight measurement and medical assessments (12 lead ECG, ECHO, blood sampling and 24 h continuous ECG monitoring) were carried out. Once the examinations were completed, the respondent was allowed to begin the process of rehydration and food intake. There were no changes in protocol.

## 3. Results

After P1, the wrestler lost 5.15% of BW in 3 days. Laboratory analyses during the experiment are presented in Table 2. The RWL resulted in lower blood glucose levels and higher urea and uric acid levels as a sign of dehydration and higher levels of AST and LDI. High levels of myocyte enzyme damage (increased CK) were reported after P1 and P2, including MBI after P2. Troponin levels remained the same, but NT pro-BNP was higher after the RWL when compared to baseline and P2. Creatinine levels were increased after P2.

After P1 and P2 complete blood count showed substantially increased leukocytes and neutrophils count, with slightly declined lymphocytes.

Echocardiography after P1 and P2 showed preserved systolic and diastolic function and no signs of ischemia or myocardial injury with preserved left ventricular global longitudinal strain.

**Figure 2:**
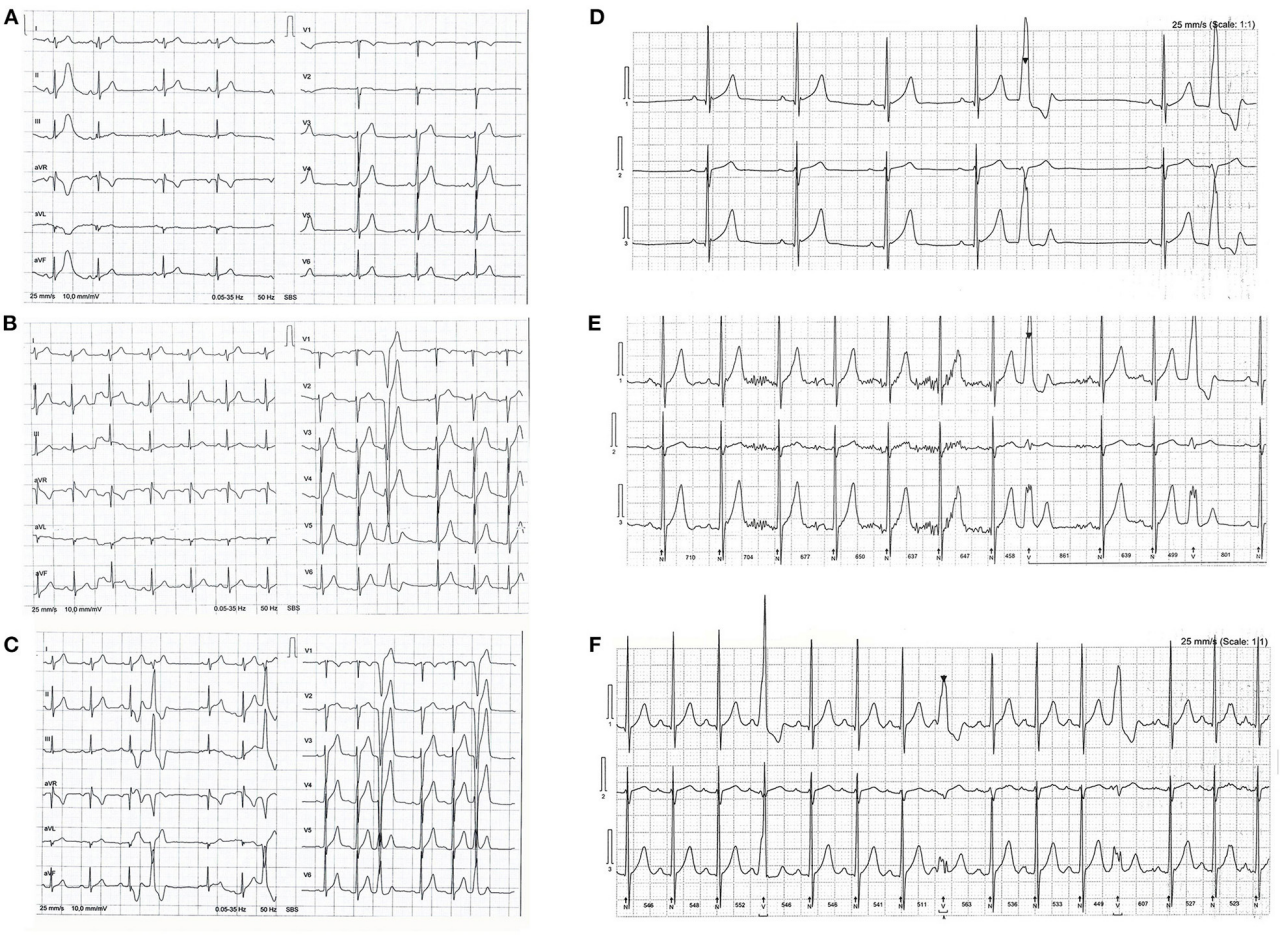
**(A)** Shows a baseline electrocardiogram (ECG). Sinus rhythm and one ectopic premature atrial beat. **(B)** Shows an ECG after P1, phase one (rapid weight loss & high intensity training); sinus rhythm and one ectopic premature ventricular beat (PVB). **(C)** Shows an P2, phase two (high intensity training) ECG with sinus rhythm and PVB in trigeminy. Three lead ECG Holter monitoring after P1 shows ectopic PVB in bigeminy in **(D)** and trigeminy in **(E)**, frequent ectopic PVB after P2 in **(F)**.

Electrocardiograms are presented in Figure 2. Baseline ECG showed sinus rhythm, heart rate (HR) of 52 beats per minute (bpm), and one ectopic premature atrial complex (PAC). After P1, ECG revealed sinus rhythm with one ventricular premature beat (VPB). Continuous 24 h ECG monitoring revealed the sinus rhythm with an average heart rate HR of 79/ bpm, minimum HR of 46/bpm, and maximal HR of 131/bpm, with frequent 2150 VPB in total for 24 h, with 2 couplets, and 8 PAC. As a therapeutic measure, a period of de-training was recommended. After 4-week period, 24 hours of continuous ECG monitoring revealed practically normal reports: sinus rhythm, average HR 68/bpm, maximal 101/bpm, and minimum 36/bpm, with occasional 244 VPB, 883 PAC, 39 in couplets, 39 times in salvos no longer than 4 beats. After P2, 24 h of ECG monitoring revealed sinus rhythm, maximal HR 126/bpm, min 49/bpm, average 79/min, with even more frequent VPB, 5% of all beats for 24 h, 4205 in total, almost all VPB was in bigeminy and trigeminy. Although the athlete was asymptomatic, he was advised against RWL and extremely vigorous exercise and for regular, frequent checkups with ECG monitoring during and after exercise. On the first check-up after 3 months, he was asymptomatic and engaged in a regular training routine.

## 4. Discussion

We reported a case of a male wrestler who developed disrupting arrhythmia during RWL and HIT. Physiological adaptation of an athlete's heart might be related to arrhythmic expression, plus certain procedures like RWL and HIT can pose an additional risk. The magnitude of the problem is unknown because there is no regular screening during these procedures.

Problematic RWL practices in wrestling were documented in the scientific literature nearly 100 years ago (10). In the wake of the tragic deaths of young wrestlers in the USA, the conversation around the health and wellbeing of combat sports athletes intensified, and the American College of Sports Medicine (ACSM) published an updated position statement on weight loss in wrestlers (11) while the National Collegiate Athletic Association (NCAA) introduced a Wrestling Weight Certification Program in 1997 (12) in the attempt to prevent extreme weight making in this sport. The newly introduced rules (i) limited weight loss to ≤ 1.5% of body weight per week; (ii) established a minimal competitive weight for each wrestler based on a lower limit of 5% body fat (males); (iii) moved weighins to 1 to 2 h pre-competition; (iv) added 3.2 kg to each weight category limit; (v) banned the use of unsafe RWL methods; (vi) randomized the order of weight class competition (instead of heavier athletes competing later), and (vii) obligated athletes to pass a hydration test (urine specific gravity ≤ 1.020) at the weigh-in.

Features of RWL (mainly ionic disturbances) and high-intensity training (high adrenergic tone, high ventricular wall stress) may predispose ischemia and arrhythmia. The authors (13) found an increase in heart rate observed as higher sympathetic modulation after RWL and concluded a higher cardiovascular risk in athletes as a result of RWL. The features of RWL affect the hemodynamics of the cardiovascular system, provoke plasmatic volume decrease, increase rest heart rate, lowers blood ejection, and finally result in a decreasing capacity to sustain work at a constant rate (11). In our case report athlete has experienced lower blood glucose levels after P1, this could be largely due to significantly lower energy intake during RWL. We can see that glucose levels after P2 are similar to baseline levels. Exercise is known to improve insulin sensitivity, although an increase in catecholamines might result in hyperglycemia just post-intense exercise (14).

Abnormally elevated values of blood urea nitrogen and uric acid were measured after P1. Creatinine is increased after P1 but even more, above the reference range after P2. Creatinine levels may increase after intense exercise as a result of exercise-induced muscle breakdown (15). A systematic review of 10 studies (16) found that creatinine, blood urea nitrogen, and urine-specific gravity values were significantly increased after RWL in the majority of the included studies. In a study by Cicioglu et al. (17) a decrease of −5.30% in total body water was reported after RWL. However, dehydration is present also in prolonged exercise. In a study by Bongers et al. (18) subjects were dehydrated on average 0.6 ± 0.3% and 2.9 ± 0.7% after acute and prolonged exercise, respectively (*p* < 0.001). Dehydration during RWL and subsequent acute kidney damage despite various degrees of weight loss characterize RWL. In addition, strength prolonged exercise accompanied by dehydration activates renin–angiotensin–aldosterone system which in these conditions may result in ischemic kidney injury (19). The estimated glomerular filtration rate significantly decreased after prolonged exercise (18). In another study (20), a significant correlation between creatinine and reduced lean body mass during RWL was observed.

Athlete's exhibited 2-fold increased CK after P1 and almost 3-fold after P2 in addition to elevated MBI above the reference value. CK and LDI are fragments of myosin heavy chain and are related to muscle damage. It is known that intense exercise leads to muscle tissue injury causing CK to be released into the bloodstream (21). Vigorous exercise results in elevations of plasma MBI in a significant proportion of athletes. Increases in these enzymes are not considered to be associated with myocardial injury but rather with muscle damage. Marked increases in CK activity are often associated with an increase in AST associated with higher muscular activity (22).

Troponin levels remained the same, but NT pro-BNP was higher after the RWL when compared to baseline and P2, though did not exceed reference ranges. NT-proBNP are mostly are released from cardiac chambers in response to volume or pressure overload and myocardial strain (23). Elevation in this biomarker can be early marker of imposed stress on myocardium during RWL. But further research in this field is needed.

Physical exercise may affect changes in the immune system. In a study that included 800 healthy young individuals exercise significantly increased white blood cells, segmented neutrophils, band neutrophils, eosinophils and to a lesser extent lymphocytes (24). In a study by Shariat et al. young judoists experienced significant increase in white blood cell count after RWL. Leukocytes after P1 follow 2-fold increase when compared to P2. Psychophysical stress during RWL could contribute to significant increases in cortisol and testosterone levels, that could additionally increase and mobilize leukocytes during RWL.

Cardiac remodeling poses the risk of increased arrhythmias at the atrial and ventricular levels. The concept of the athlete's heart as a proarrhythmic has been described previously (9). In a large-scale study by Spirito et al. (25), the findings indicated that wrestling has a high impact on LV wall thickness size but a relatively small impact on cavity dimension, suggesting that wrestling has a disproportionate influence on wall thickness relative to cavity dimension, which is comparable with our study results. In a similar fashion, Cohen and colleagues (26) found the left ventricular posterior wall, interventricular septal, and left ventricular internal dimensions to be significantly larger in high school wrestlers who had seasonal variations in the weight of 9% than in non-athletic controls during systole (*p* < 0.05) and diastole (*p* < 0.025). Left ventricular remodeling might promote arrhythmia in these athletes. Continuous vigorous exercise may result in right heart remodeling and cardiac structure and function alteration (27). Sustained volume overload in endurance sports may result in acute right heart chambers dilatation and chronic replacement of the myocardium with fibrotic areas that could be a substrate for ventricular arrhythmias (28). Although wrestling is categorized as a strength sport, many sports include frequently overlap between isotonic and isometric exercise, and thus pose mutual risks. Differentiating physiological responses following sport participation from pathology conditions could be challenging. Characteristics of exercise-induced remodeling may resemble very early features of arrhythmogenic right ventricular cardiomyopathy (ARVC), inherited cardiomyopathy linked to fatal arrhythmias (29), and sometimes it is difficult to differentiate between these two entities (27). In these individuals, cardiac magnetic resonance (CMR) a powerful imaging technique may enhance the evaluation of cardiac structure and function (30) and help in the differentiation between physiological and pathological conditions. In a study evaluating the overlap between typical features of ARVC and sport-related peculiarities by CMR, 16% of young athletes had abnormally increased RV volumes, one of the diagnostic criteria for ARVC. However, the diagnosis of ARVC is complex and involves subsequent diagnostics and fulfillment of the Task Force Criteria (TFC) (31). In a study investigating ventricular arrhythmias in 4,263 athletes, the prevalence of mostly isolated frequent and monomorphic ventricular arrhythmias was 4.19% in a healthy heart without structural cardiomyopathy, with the mean daily number of 1,101 +/−2,693 (range 0–16,678) (32). An increasing number of PVBs, specific morphology (site of origin), and characteristics should raise the red flag for investigation in underlying heart disease. There is no absolute threshold in athletes in the number of PVBs that is used as a cut-off for underlying disease. Biffi (33) et al. found that >2,000 PVCs per day in asymptomatic athletes were associated with a 30% of chance of finding an underlying structural or cardio-genetic disease. A meta-analysis of ten studies found that PVB in the recovery phase of an exercise test, not during exercise, was correlated with a higher risk of adverse cardiovascular events in the long term (34).

In another study, elite athletes with ventricular arrhythmias during exercise had evidence of impaired RV function, myocardial fibrosis, and additional LV contraction abnormalities in life-threatening arrhythmic events. The authors concluded that ventricular arrhythmias are more commonly associated with cardiovascular abnormalities in young competitive and female athletes and if present, they require a thorough investigation and follow-up. They postulated that these phenotypes imitate arrhythmogenic cardiomyopathy and may potentially be provoked by vigorous exercise in susceptible individuals (35).

## 5. Conclusion

**Figure 3:**
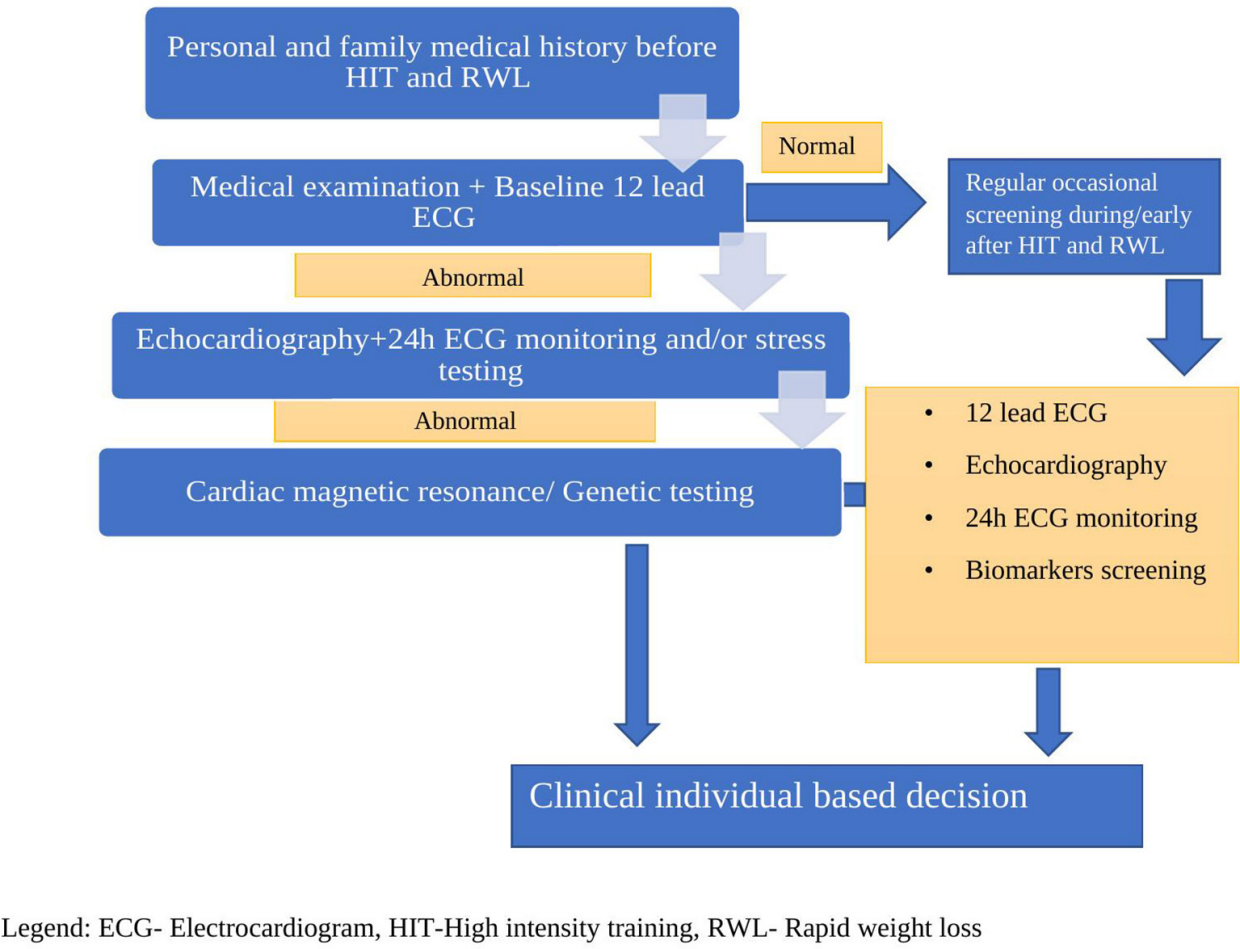
Suggested cardiovascular screening for athletes engaged in HIT and RWL.

We know that physiological adaptation and heart remodeling can yield the risk of arrhythmia, especially in susceptible individuals, whilst regularly practiced methods like RWL and HIT pose the additional risk that may possibly confer unfavorable prognosis even in asymptomatic individuals. We postulate that cardiovascular screening is necessitated, especially during high-risk methods like RWL and HIT, in helping us prevent adverse outcomes and come to individual-based clinical making decisions (Figure 3).

### 5.1. Limitations

This study is single case report which *per se* has its limitation as lack of ability to generalize, potential of over interpretation etc. Although the research in this area is scarce, preliminary conclusions and hypothesis that screening is necessitate part of different procedures the athletes is involved may highlight the need for research in this area.

## Data availability statement

The original contributions presented in the study are included in the article/supplementary material, further inquiries can be directed to the corresponding authors.

## Ethics statement

The studies involving human participants were reviewed and approved by Ethics Committee of the University of Novi Sad, Novi Sad, Serbia (Ref. No. 46-06-02/2020-1). The patients/participants provided their written informed consent to participate in this study. Written informed consent was obtained from the individual(s) for the publication of any potentially identifiable images or data included in this article.

## Author contributions

AM, MP, and PD conceived and designed the research. RR, TM, and AI conducted experiments. ASM and TT analyzed data and interpreted the results of the experiments. AM and NL drafted the manuscript and prepared tables. All authors edited and revised the manuscript and approved the final version of the manuscript.
